# Scanning Ion Conductance Microscopy for Studying Biological Samples

**DOI:** 10.3390/s121114983

**Published:** 2012-11-06

**Authors:** Patrick Happel, Denis Thatenhorst, Irmgard D. Dietzel

**Affiliations:** 1 Central Unit for Ionbeams and Radionuclides (RUBION), Ruhr-University of Bochum, Universitätsstraße 150, D-44780 Bochum, Bochum, Germany; 2 Department of Molecular Neurobiochemistry, Ruhr-University of Bochum, Universitätsstraße 150, D-44780 Bochum, Bochum, Germany

**Keywords:** scanning ion conductance microscopy, live cell imaging, ion selective micro-electrodes

## Abstract

Scanning ion conductance microscopy (SICM) is a scanning probe technique that utilizes the increase in access resistance that occurs if an electrolyte filled glass micro-pipette is approached towards a poorly conducting surface. Since an increase in resistance can be monitored before the physical contact between scanning probe tip and sample, this technique is particularly useful to investigate the topography of delicate samples such as living cells. SICM has shown its potential in various applications such as high resolution and long-time imaging of living cells or the determination of local changes in cellular volume. Furthermore, SICM has been combined with various techniques such as fluorescence microscopy or patch clamping to reveal localized information about proteins or protein functions. This review details the various advantages and pitfalls of SICM and provides an overview of the recent developments and applications of SICM in biological imaging. Furthermore, we show that in principle, a combination of SICM and ion selective micro-electrodes enables one to monitor the local ion activity surrounding a living cell.

## 1. Introduction

Since the microscope has been invented in the 16th century, microscopes are used to study biological samples with a resolution beyond the limit of the human eye. Today, in general two different types of microscopes exist. The first and larger group comprises microscopes that operate in the far field. These microscopes use light or, in case of scanning or transmission electron microscopes, electrons to illuminate the sample and detect for example reflection, scattering or fluorescence. Since waves are used to illuminate the sample, these microscopes are subject to the diffraction limit that has been described by Ernst Abbe and that links the resolution of the microscope to the wavelength of the radiation used to illuminate the sample [1]. Nevertheless, recent developments in far-field microscopy allow to utilize certain physico-chemical properties of fluorescent molecules to circumvent Abbe's limit [2–6].

The second group of microscopes comprises the scanning probe microscopes. These microscopes use a tiny probe to measure a physical variable that depends on the distance between the probe and the sample surface. The first scanning probe microscope, the scanning tunneling microscope, was introduced in 1982 by Binnig, Rohrer and co-workers. It uses the tunneling current between a conducting probe and a conducting surface to determine positions of equal distance between tip and surface and thus to reconstruct the topography of conducting samples [7]. This technique, which was awarded with the Nobel Prize in 1986, requires the sample to be conductive and furthermore operates in vacuum, and hence can only image biological samples in an artificial environment after complex sample preparation. The second scanning probe technique developed by Binnig, Rohrer and their colleagues, the atomic force microscope (AFM) [8], utilizes the deflection of a soft cantilever with a sharp cone at its end that is dragged over the sample and hence senses its surface structure. Although the strengths of the AFM are the imaging of hard samples at even sub-atomic resolution [9], it had been quickly adapted to image biological samples [10–13]—the state of the art of AFM-imaging of biological samples has been reviewed recently [14]. Despite these successful recordings, the force which is applied by AFM to the sample cannot be neglected [15,16] and hence imaging living cells without biasing still remains a challenging task.

In 1989, Paul Hansma and colleagues invented the scanning ion conductance microscope (SICM) [17]. It monitors the ionic current through the tiny opening of an electrolyte filled glass micro- or nano-pipette. The current flow through the opening is hindered if the tip is in close proximity to a non-conducting surface. This relation between ionic current and tip-sample distance is utilized to determine the topography of the sample, either by calculating the distance between probe and sample from the current flowing through the tip opening, or, more often, by determining positions of equal resistance change which are used to reconstruct the sample topography.

**Figure 1.**
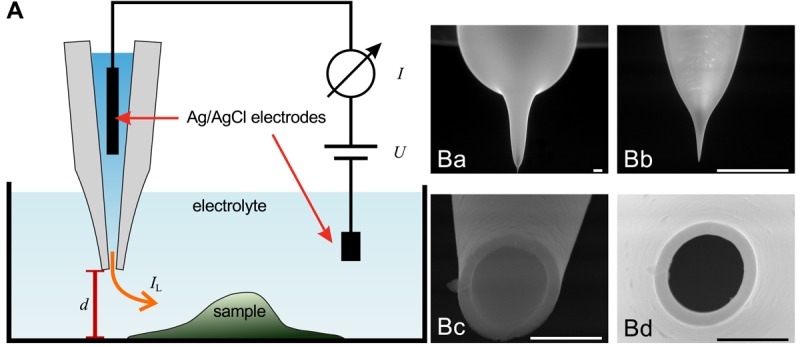
(**A**) Schematic illustration of the setup of a SICM. A voltage is applied between two Ag/AgCl-electrodes, one of which is located within the scanning pipette (gray). The two electrodes are connected via an electrolyte solution. The ionic leakage current *I*_L_ through the tiny opening of the pipette depends on the distance *d* between pipette and sample; (**B**) Scanning electron micrographs of a scanning pipette. Scale bars: 100 *μ*m (Ba, Bb) and 1 *μ*m (Bc, Bd). Note, that the images in Ba–Bc contain a perspective distortion due to a non-perpendicular imaging angle.

The general assembly of a SICM is similar to electrophysiological setups, as shown in Figure 1A. A voltage is applied between two Ag/AgCl-electrodes, one of which is located in bulk electrolyte solution, the other one is inserted into a glass pipette containing the same electrolyte solution. The leakage current *I*_L_ through the opening of the pipette depends on the distance *d* between probe and sample surface.

The mathematical descriptions of an ionic current through the aperture of a micro-pipette base on the description of the Scanning Electrochemical Microscope by Bard and colleagues [18,19] that was adapted to SICM by the group of Harald Fuchs [20]. The different approaches and approximations to describe the current through a pipette depending on the shape of the pipette have been reviewed in detail recently [21], hence we focus on the description of the imaging process for a given pipette.

Scanning electron micrographs of a scanning probe are shown in Figure 1B. The probe consists of a bulk region (top in Ba) and two tapering regions, the second one magnified in Figure 1Bb. The opening diameter of this pipette approximated one micrometer (Figure 1Bc,Bd). If combined with physiological bath solution, probes with this geometry have an access resistance in the range of 4 MOhm to 6 MOhm. A pipette like this one allows recordings with a resolution in the micrometer range. However, pipettes with a smaller opening diameter allow recordings with resolutions in the nanometer range. Using nano-pipettes with an opening diameter of 13 nm a spatial resolution in the range of 3 nm–6 nm has been observed [22]. However, the resolution of SICM still is a matter of debate, since in contrast theoretical considerations suggest a resolution of 3/2 inner pipette diameters [23].

**Figure 2.**
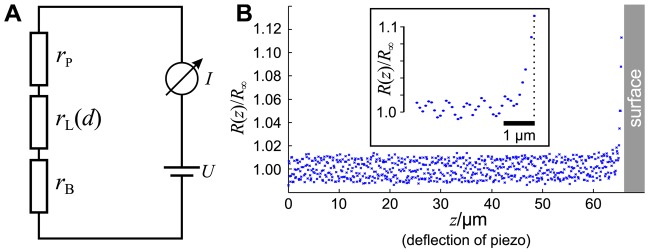
(**A**) Circuit diagram of a SICM. The current flows through the three resistors *r*_P_, *r*_L_(*d*) and *r*_B_. The resistance of *r*_L_(*d*) depends on the distance *d* between sample and probe and is utilized to determine the topography; (**B**) Typical resistance-distance or approach curve. After an approximately steady resistance at large tip-sample distances, the relative resistance *R*(*z*)/*R*_∞_ increases with when the tip approaches the sample surface. The inset shows a magnification of the last micrometers.

As shown in Figure 2A, the circuit of a SICM can be described by a series of three resistors. The resistors *r*_P_ and *r*_B_ denote the resistances of the pipette and the bulk solution, respectively, and depend both on the conductance of the electrolyte as well as on the position and shape of the Ag/AgCl-electrodes (see [21] for review). The resistance *r*_L_ of the leakage current *I*_L_ through the aperture of the electrode depends, besides the shape of the tip, on the opening diameter and the distance *d* between the aperture and the sample surface [20,23,24].

The total resistance *R* of the setup is the sum of these three resistances. Since *r*_L_ is a function of the distance *d* between tip and sample, the total resistance also depends on *d*:

(1) $$R(d)=r_{\text{P}}+r_{\text{L}}(d)+r_{\text{B}}.$$

Note that we neglect that the bath resistance *r*_B_ is a function of the position of the tip opening (and therefore of *d*) since it depends on the distance between tip opening and bath electrode. Furthermore, we neglect the voltage drop at the surfaces of the AgCl-electrodes, assuming they are constant and much smaller than the voltage drop at the pipette tip. Furthermore, we denote the reference resistance of the entire system at infinite tip-sample distance *R*(*d* = ∞) = *R*_∞_.

A typical approach curve recorded with a micro-pipette as shown in Figure 1B is shown in Figure 2B. Note that since the initial distance *d*_0_ between tip and sample is unknown, the deflection *z* of the piezo is recorded instead. The distance is the difference between the initial distance *d*_0_ and the piezo deflection *z: d* = *d*_0_ − *z*. While the resistance *R*(*z*) of the system matches the reference resistance *R*_∞_ as long as the tip-sample distance is large and hence *R*(*z*)*/R*_∞_ ≈ 1, the resistance increases asymptotically as the pipette tip approaches the surface (hence *z* → *d*_0_ and *d* → 0).

A normalized approach curve as shown in Figure 2B can be approximated as

(2) $$\frac{R(d)}{R_{\infty }}=1+\frac{D}{d}=1+\frac{D}{d_{0}-z},$$

where *D* denotes the tip-sample distance that doubles the resistance [20,25]. The parameters *d*_0_ and *D* can be determined by numerically fitting this equation to the data. *D* is determined by the geometric properties of the electrode and the electrolyte. Basing on *D*, every distance *D*_Δ_*_T_* at which the resistance has increased by a certain amount Δ*T* can be determined by calculating *D*_Δ_*_T_* = *D*/Δ*T*. Since often recordings of approach curves end before the resistance is doubled, it might be more successful to directly fit

(3) $$\frac{R(d)}{R_{\infty }}=1+\Delta T\frac{D_{\Delta T}}{d}=1+\Delta T\frac{D_{\Delta T}}{d_{0}-z}.$$

**Figure 3.**
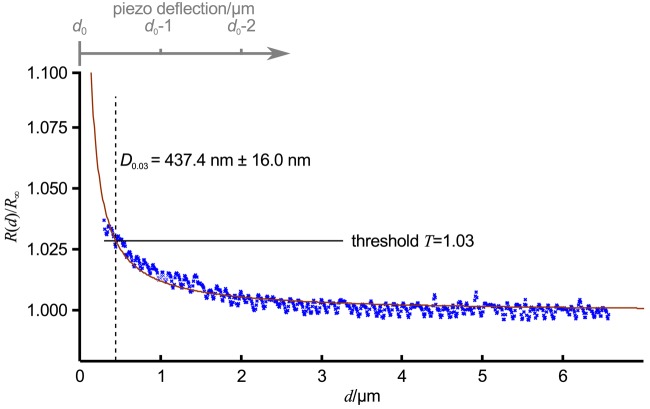
Approach curve recorded with a typical SICM probe (blue crosses), data was low-pass filtered by applying a first order Butterworth filter with a cut-off frequency of 1/*μ*m after acquisition. Red line shows a fit of Equation (3) to the data, Δ*T* was selected as 0.03, hence the threshold *T* was 1.03 (solid black line). With these settings, a pixel would be detected at a tip-sample distance of 437.4 nm ±16.9 nm (dashed black line). The gray axis indicates the piezo deflection.

Figure 3 shows an approach curve with Equation (3) fitted (red line) to the low-pass filtered data (blue crosses) of an approach curve, Δ*T* was chosen as 0.03 (indicated as relative threshold *T* = 1 + Δ*T* by the black line). For the given pipette, which was similar to the one in Figure 1B, *D*_0.03_ was 437.4 nm ± 16.0 nm (dashed black line). Note that here the approach curve was plotted versus the tip sample distance *d*, the piezo deflection is indicated by the gray axis at the top of the diagram. The resistance starts to increase approximately at a tip sample distance of 2.5 *μ*m, that is approximately 2.5 inner diameters of the pipette. Nevertheless, a distinct signal that can be clearly distinguished from the remaining noise appears at approximately 1.5 *μ*m (and hence 1.5 pipette diameters).

## 2. Imaging Modes of SICM

**Figure 4.**
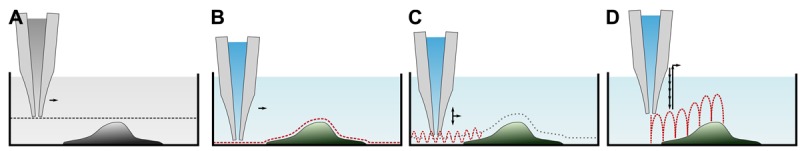
Imaging modes of SICM: (**A**) The pipette is moved laterally at a constant *z*-position and the resistance is monitored from which the topography is calculated (constant *z* mode); (**B**) The pipette is moved laterally and a feedback loop modulates the *z*-position of the pipette until a predefined resistance establishes. The topography is determined by the *z*-position of the pipette (direct current (DC) or constant distance mode); (**C**) While the pipette is moved laterally, the *z*-position is modulated sinusoidally. In close proximity to the sample, the resulting resistance is sinusoidal, too. The amplitude of the sinusoidal resistance serves as a feedback that modulates the average *z*-position until a predefined amplitude establishes. The average *z*-position is used to represent the topography (alternating current (AC) mode); (**D**) The pipette is lowered towards the sample and the approach is stopped when a predefined resistance is reached. The pipette is then dragged back, moved laterally and the process repeats (note that the trajectory of the probe is depicted parabolically for clarity). The *z*-positions at which the approach has been stopped represent the sample topography (backstep or hopping or standing approach (STA) mode).

Since its invention, various ways to determine the sample topography with a SICM have been developed. A brief overview of the imaging modes is provided in Table 1. Although seldom used for imaging, let us start with a general scan mode as depicted in Figure 4A first. Here, the pipette is lowered towards the surface until a resistance change is detected. The pipette is then moved laterally while maintaining its *z*-position and monitoring the resistance. Assuming a constant conductivity of the electrolyte solution, the distance *d* between tip and sample can be calculated using Equation (2). However, the distance range in which the probe senses the sample is small (see Figure 3), hence only flat samples with small variances in height can be imaged.

### 2.1. Modes Modulating the Pipette Position

All other modes differ from this mode since they alter the *z*-position of the scanning probe during the scan until a predefined threshold value establishes and record the probe position instead of the resistance. These modes assume a constant resistivity of the sample surface, hence the change in resistance only depends on the tip-sample-distance (see Figure 3). Coordinates of equal resistance change are approximated to represent the sample topography.

The first mode, which was already published in the first report of SICM [17], is depicted in Figure 4B. Once the probe is approached to the sample, the difference between the present resistance and a predefined threshold resistance is used as a feedback signal that modulates the *z*-position of the probe until the difference is zero. This mode utilizes the direct current to maintain the tip-sample distance, and hence is called DC or constant distance mode.

Since the modulation of the *z*-position of the scanning probe follows its lateral movement, abrupt differences in the sample height larger than the tip sample distance inevitably lead to physical contact between tip and sample. This either destroys the sample or the probe or at least pushes or dents the sample. To partially circumvent this problem, imaging at large tip sample distances has been suggested [17,26]. However, large tip sample distances reduce the sensitivity of the SICM [20,23,27]. Furthermore, this mode is very prone to DC drifts, for example due to unstable potentials at the surface of the Ag/AgCl-electrodes.

A major improvement has been made by the superimposition of the DC signal with an alternating, sinusoidal current component (in the range of 1kHz–2 kHz) [28,29] and hence is called AC mode (similar to the AFM tapping mode). If the vibrating probe is in close proximity to the surface, the signal comprises both a DC and an AC component. The amplitude of the latter one is detected by a lock-in amplifier and serves as a feedback signal that modulates the *z*-position of the probe. The major advantage is that the response of the AC component increases much steeper than the DC component and hence the surface can be detected at larger distances. Furthermore, the AC component is less prone to DC drifts, and, in combination, the modulation amplitude of the probe (in the range of a probe diameter) and the sensing of the surface at larger distances enable recordings of steeper slopes and more complex samples.

**Figure 5.**
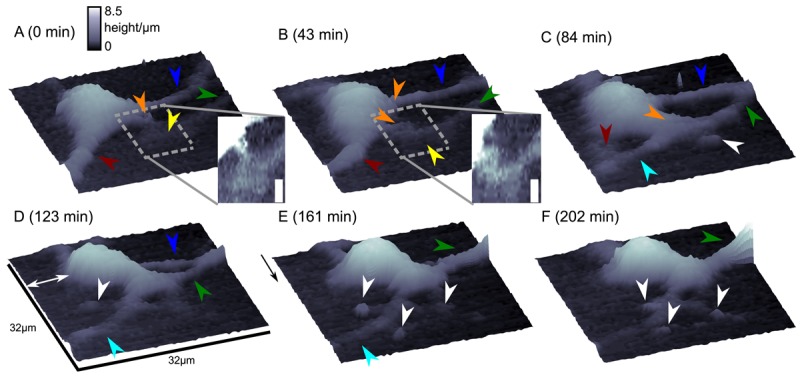
Long term imaging of a living neural cell from rat brain. Images were obtained in backstep (hopping/STA) mode at times as indicated. Lateral step size was 500 nm, vertical step size was 100 nm, imaging time was approximately 30min per image. Image size indicated in D, arrow heads mark several rearrangements of cellular processes, insets show magnifications of the marked areas with increased contrast, scale bar: 3.5 *μ*m. Figure taken from [35], licensed under a creative commons license (http://creativecommons.org/licenses/by/2.0/).

Although this mode enables stable recordings of samples of moderate sample topography, it is not possible to image steep and abrupt slopes as frequently occur at cell borders. This limitation has been overcome by introducing a non-continuous mode that retracts the scanning probe after the surface has been sensed, positions the probe laterally and approaches the sample again to detect the succeeding pixel. Similar modes have been known from the generation of force distance maps using AFM [30,31]. For SICM, this mode has been first published by Stefan A. Mann from our group [32], using the term backstep mode of pulsed SICM. After introducing further refinements, Novak and colleagues succeeded in the recording of high-resolution images under the name Hopping Probe Ion Conductance Microscopy (HPICM) or, more often used today, hopping mode [33]. Furthermore, mostly if combined with scanning electrochemical microscopy, the term standing approach (STA) mode is used [34]. Despite the use of different names, all scanning procedures using these modes feature the same advantages and pitfalls. First of all, since the probe is retracted after every pixel, large differences in height can be imaged easily without impairing the sample. Figure 5 shows a long term recording of a live neural cell from rat brain obtained in backstep (hopping/STA) mode (taken from [35]).

**Figure 6.**
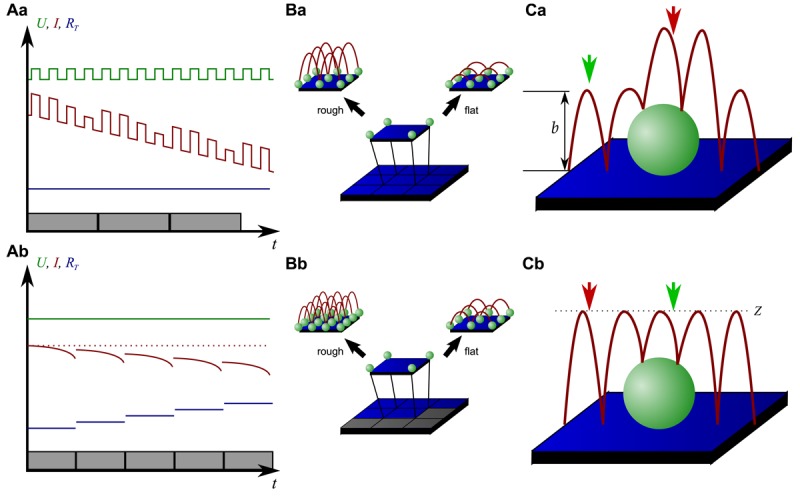
Improvements of backstep mode. (**A**) shows two methods to circumvent the effect of DC drifts, either by applying pulses (**Aa**) or by adapting the reference resistance (**Ab**). (**B**) shows improvements of the temporal resolution by changing the retraction distance (**Ba**) or both the retraction distance and the resolution (**Bb**) after a low resolution pre-scan. (**C**) shows two methods of pipette retraction, either by a pre-defined distance (**Ca**) or to a pre-defined position (**Cb**). Red arrows indicate regions that require long approaches of the probe, green arrows mark regions that require only short approaches.

On the other hand, since the recording of every pixel requires an entire approach curve over a relatively large distance, scanning speed is inevitably slow. For example, the images in Figure 5 required approximately 30 min each. Furthermore, since the backstep (hopping/STA) mode relies on the direct current signal, it is also prone to slow DC drifts. The latter problem has been already addressed in the paper that introduced the backstep mode [32] by applying voltage pulses of defined height instead of a constant voltage as displayed in Figure 6Aa. Instead of measuring the current, the pulse height is determined that is independent from slow DC drifts. Another way to circumvent the impact of slow DC drifts is the determination of the reference resistance at the beginning of every single approach [33]. In this case, the reference resistance adapts to the DC drift during the image acquisition (Figure 6Ab).

To increase the temporal resolution the usage of a low resolution pre-scan of the sample has been introduced [36]. The height differences representing the roughness of the sample are analyzed and the distance by which the scanning probe is retracted is adjusted accordingly (Figure 6Ba). Assuming a sample with steeply increasing slopes covering 20 % of the entire image and a reduction in retraction distance of 75 % in the remaining area, the image acquisition time is reduced by 60 %.

Figure 6Bb shows further improvements that have been introduced by Novak *et al.* [33]. First, the time between the preceding low resolution scan and the subsequent high resolution scan was reduced. After obtaining a square of four pixels at low resolution, the roughness of the corresponding topography was analyzed immediately and subsequently the corresponding area imaged at high resolution. This is particularly useful for imaging living samples such as cells that frequently undergo changes in shape since it ensures that the low-resolution data is still valid and the topography of the currently imaged area has not changed between pre-scan and high resolution scan.

Furthermore, acquisition time was reduced by not only adjusting the retraction distance, but by adapting the imaging resolution, assuming that flat areas comprise areas of less interest (most likely the bottom of the recording chamber).

Figure 6C shows two different ways of retracting the scanning probe during image acquisition. Either, the probe can be retracted by a pre-set distance (*b* in Figure 6Ca) or to a pre-set *z*-position (*Z* in Figure 6Cb). While in the first mode a smaller retraction distance might be selected and hence shorter approaches might occur at the lower areas of the sample (green arrow in Figure 6Ca), approaches of the same distance occur at the upper areas of the sample (red arrow in Figure 6Ca). In the latter mode, long and short approaches occur opposed: Since the approach distance is the difference between the sample height and the selected *z*-position, shorter approaches are required at the higher areas of the sample (green arrow in Figure 6Cb), whereas larger approaches occur at lower sample areas (red arrow in Figure 6Cb).

Very recently, a new scanning mode has been introduced which dramatically decreases the scanning time while it maintains high resolution [37]. This mode, called FSICM (fast SICM), combines the advantages of DC and backstep (hopping/STA) mode and furthermore uses the current determined during scanning to reconstruct the surface, thus being a hybrid method of the methods in Figure 4A,B and C.

First, a rough estimation of the very first line of the sample is obtained by backstep (hopping/STA) mode. The topography of this line is then refined by scanning the pipette along this estimation, while recording the current through the probe opening. The current then is used to reconstruct a more detailed surface image for that particular line. This is repeated several times to determine an exact topography, which is used as the reference for the succeeding line. This mode allows scanning with much higher pixel densities (the authors report 1,024 × 600) in sub-minute temporal resolution of a sample that, imaged in backstep (hopping/STA) mode, requires approximately 25 min at a pixel density of 128 × 128. However, since this method relies on the similarity of adjacent lines of the sample, abrupt changes in height that occur perpendicular to the scanning direction might lead to collisions of the probe with the sample.

### 2.2. General Methods to Determine the Resistance during SICM Scanning

In general, the resistance *R*(*d*) of an SICM in dependence of the probe-sample-distance can be determined by either applying a constant voltage and measuring the current or by adjusting the voltage until a constant current flows. Both methods are known from various scientific fields, called either voltage clamp (VC) or current clamp (CC) in electrophysiology, or potentiostat and galvanostat in electrochemistry. Most set-ups use the VC approach, since a constant voltage does not cause capacitive currents. However, CC mode amplifiers are required if the recording of a second signal requires the determination of a potential, like e.g., measurements with ion selective electrodes. Hence, in order to treat both methods, in this review we refer to the resistance which comprises both methods connected by Ohm's law.

## 3. Imaging Biological Samples with SICM

**Figure 7.**
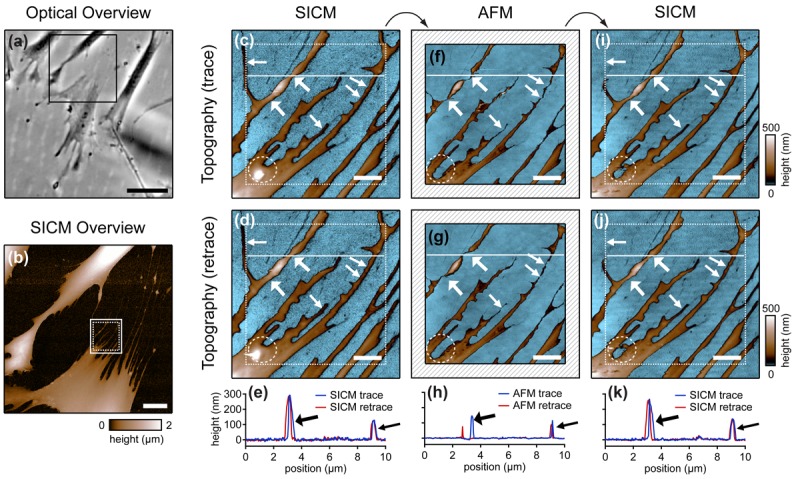
Comparison of SICM and AFM in AC- and tapping-mode. Extensions of a fixed fibroblast (box in **b**, corresponding phase contrast image in **a**) were imaged both with SICM and AFM repeatedly, an SICM overview image is provided in (**b**). (**c**, **f**, **i**) Topography images obtained by SICM (**c**), AFM (**f**) and again by SICM (**i**) show that small objects appear thinner and lower in the AFM image (small diagonal arrows). Bold white arrows mark regions less attached to the substrate that are shifted towards the scanning direction by AFM, compare the retrace scan (**g**, opposite scanning direction) and the corresponding profiles (**h**). In contrast, SICM traces and retraces match. The horizontal arrow and the dashed circle mark areas that potentially have been destroyed by the AFM scan, since they are not visible in the second SICM recording. Reprinted with permission from [38]. Copyright 2011 American Chemical Society.

Cells are soft, delicate samples that are easily affected during the imaging process. Even if they are fixed, which means that the proteins within a cell are altered chemically such that their biochemical activity is arrested and the cells are not considered as living, cells remain soft and hence prone to distortions by the scanning probe. Rheinlaender and colleagues compared the influence of the imaging process on fixed cells that were imaged both by AFM and SICM [38] in AC- and tapping-mode. They showed that in AFM images of fine cellular structures such as cell extensions, these structures appear lower and thinner than in the corresponding SICM recordings (diagonal arrows in Figure 7), verified by the comparison of AFM and SICM images of collagen fibrils and chromosomes [39]. Furthermore, by comparing images recorded with opposite scanning directions, they showed that parts of the cell membrane that were only attached loosely to the substrate are shifted towards the scanning direction by AFM and hence appear at different positions (bold arrows and panel h in Figure 7). In contrast, in SICM recordings the locations of those loose structures were independent from the scanning direction (e and k in Figure 7).

Besides these distortions, AFM also irreversibly destroyed parts of the sample, indicated by the horizontal arrow and the dashed circle in the images shown in Figure 7. While these structures were imaged by SICM repeatedly without notable differences (Figure 7c,d), they were not observed by AFM (Figure 7f,g). A SICM scan succeeding the AFM scans did not show these structures either (Figure 7i,j), suggesting that these structures have been destroyed by the AFM imaging process.

**Figure 8.**
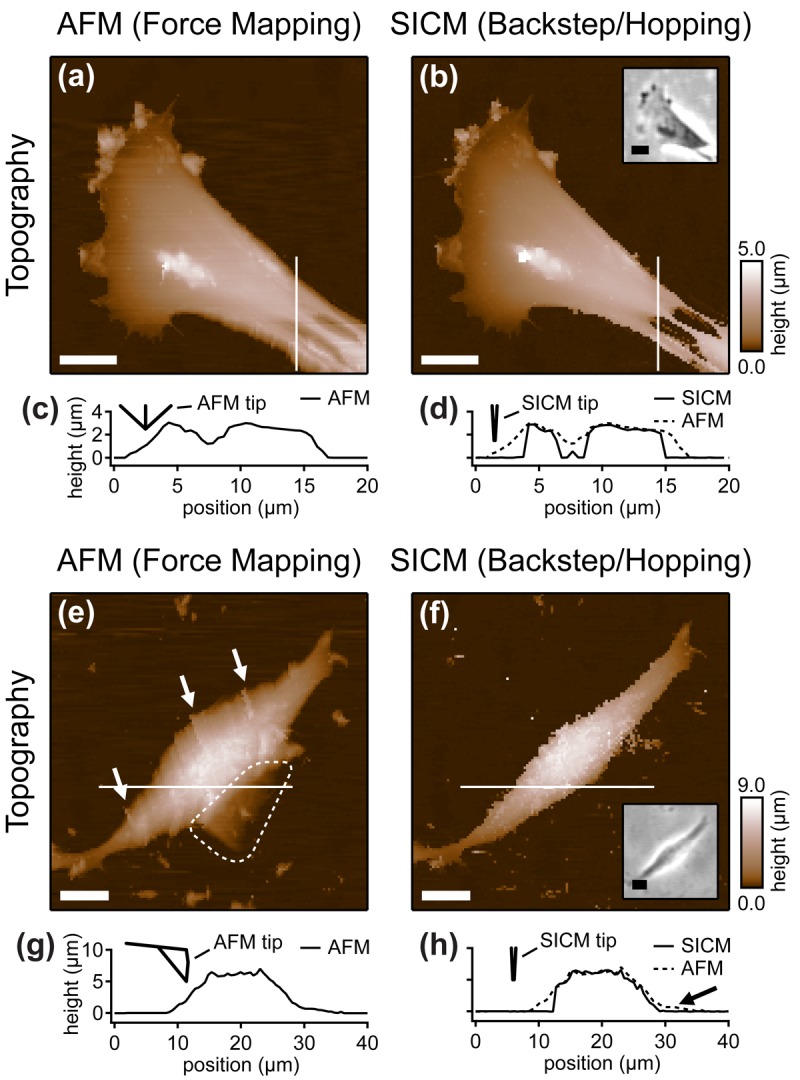
AFM force mapping (**a**, **e**) and backstep (hopping/STA) SICM (**b**, **f**) images of the two samples. The profiles along the lines in the respective scans are shown in (**c**), (**d**), (**g**) and (**h**). Note that at steep sample regions the AFM images appear broader than the SICM images. Furthermore, due to interactions between the basal part of the AFM tip and the sample, artificial tail-like structures are observed (dashed region in (**e**)). Reprinted with permission from [38]. Copyright 2011 American Chemical Society.

Furthermore, the authors compared the vertical approach modes of both scanning probe techniques: force mapping and backstep (hopping/STA) mode. They found that due to the shape of the AFM tip, the basal part of the tip interacts with the sample at steep slopes. This results in artificial topography signals at steep, narrow structures. In contrast, due to the aspect ratio of the probe, SICM allows the recording of these structures (Figure 8a–d). Additionally, the quality of SICM images has been validated by comparing SICM and scanning electron microscopy images [39].

When even higher samples are imaged, the interaction of the basal part of the tip results in the observation of artificial, tail-like structures (dotted region in Figure 8e) that are not observed with SICM (Figure 8f). Furthermore, due to the interaction of AFM tip and sample, the slopes of the sample are smoothed and therefore the sample appears broader than in the corresponding SICM recordings (Figure 8g,h).

In contrast to fixed cells as investigated above, living cells maintain parts of their physiological activity even in culture—as generally used for SICM imaging—and hence, depending on the cell type, migrate, change their shape, contract or show electrical activity. Therefore, imaging living cells is a complex task. The first SICM recordings of living cells have been reported by Korchev and colleagues [40,41]. By applying SICM to A6 cells, Gorelik and colleagues revealed the life cycle of microvilli and how a monolayer of A6 cells maintains its integrity [42–44]. SICM furthermore helped to characterize the molecular identity of the receptors mediating ATP stimulated Na^+^ channel activity in renal epithelial cells [45].

Cardiovascular cells have been extensively studied by SICM [46]. SICM recordings of cardiomyoctes allowed the definition of a new parameter, the *z-groove index*, to describe the morphology of the cell surface [47]. Furthermore, the differences in failing and healthy ventricular myocytes have been determined [48] and it has been shown, that after prolonged mechanical unloading the surface of cardiomyocytes becomes more flat [49,50].

Since SICM allows detailed, three-dimensional imaging, it has been used to investigate and characterize different cell types or stages of cellular development that can be distinguished morphologically. It revealed that corticosteroids reverse the morphologic effect of cytokines on survival and differentiation on oligodendro-glial progenitors [51]. Furthermore, SICM studies of the neuroblastoma SK-N-SH cell line showed that this cell line contains all three morphologically different cell types known from neuroblastoma [52] and supported the electrophysiological recordings from PC12 cells, which become more neuron-like after exposure to nerve growth factor [53].

SICM has been used to investigate the morphological response of endothelial cells to shear stress [54,55] and to determine the coverage of holes in an artificial membrane that was used as a substrate for astrocytes and fibroblasts [56].

Due to its resolution beyond the limit of conventional light microscopes, SICM has been applied successfully to investigate surface changes after artificially induced exocytosis and revealed that the membrane of a fraction of 16 % of the investigated cells showed tiny dips shortly after exocytosis [57]. Furthermore, SICM has been applied to determine the toxicity of various nanoparticles that induce holes in the cell membrane [58,59]. On an even smaller scale, SICM has been used to determine the movement of protein complexes within the cell membrane of living spermatozoa [22].

Although the investigations detailed above have only been possible because of the nearly non-invasive imaging characteristics of SICM, the scanning probe can be easily used to stimulate the sample. The probe can be used to apply a force on the membrane, allowing the electrophysiological recording of mechano-sensitive currents [60] or pressure applied through the scanning pipette can be used to determine the mechanical properties of the cell membrane [61,62]. Both, force by the interaction of pipette and membrane and pressure through the pipette can be used to direct growth cones of leech neurons [63,64]. Note that even the flow of electrolyte through the pipette opening that is not compensated by the capillary tension might stimulate live cells and thus impair the sample, particularly if relatively large pipettes are used.

**Figure 9.**
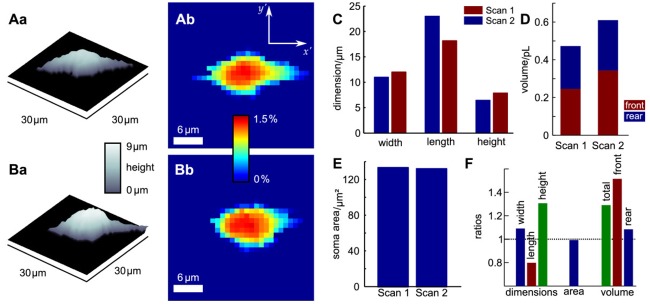
Changes of cell body dimensions of a migrating oligodendrocyte progenitor cell. **Aa** and **Ba** show three-dimensional representations of the observed cell, **Ab** and **Bb** show the approximated cell soma. **C**–**F** show the changes in cell body dimensions between the two scans. While the area of the cell remains constant, the volume increases particularly at the frontal part of the cell. Extrapolations of the volume from two-dimensional micrographs would not have detected this volume change. Figure taken from [65], licensed under a creative commons license (http://creativecommons.org/licenses/by/2.0/).

**Figure 10.**
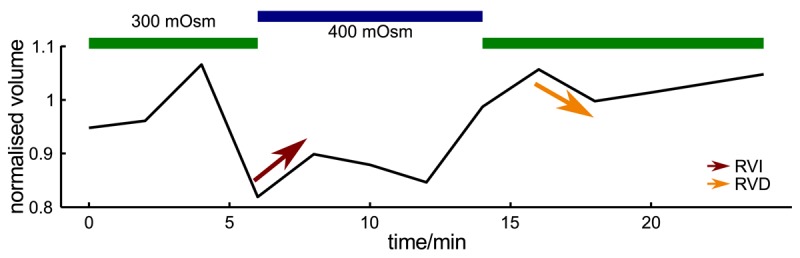
Volume changes of a rat hepatocyte after exposure to hyperosmotic solution. Hepatic cells cultured and superfused with solutions as described in [67,68]. RVI: regulatory volume increase, RVD: regulatory volume decrease. Step sizes were 2 *μ*m lateral and 100 nm vertical, probe resistance was approximately 5 MOhm.

Since probe microscopy images reveal the three-dimensional shape of a cell, not only the cellular surface but the cellular volume can be investigated with SICM [26]. Furthermore, volume changes can be attributed to specific regions of a cell [36] or, after specific processing of the data, be divided in frontal and rear volume changes even in cells that change shape and position between succeeding scans [65], as shown in Figure 9. If the resolution is decreased and a scanning time of approximately two minutes is achieved, fast volume regulatory processes can be investigated with SICM [66]. An exemplary recording is depicted in Figure 10, showing the typical fast regulatory volume increase (RVI, red arrow) after application of hyperosmolar solution and regulatory volume decrease (RVD, orange arrow) after switching back to lower osmolar control solution.

### 3.1. Combined Methods

SICM has been combined with various detection methods. For example, the scanning probe has been utilized as a light source for scanning near field optical microscopy [69,70]. For the investigation of biological samples, combinations of SICM with two major techniques have been proven particularly useful. Since SIC microscopes most commonly are build onto an inverted light microscope, this intuitively suggests to combine SICM and fluorescence microscopy. This combination was first used to simultaneously determine cellular height and intracellular calcium transients of a contracting cardiomyocyte [28]. This technique was extended to image in both lateral dimensions (and thus called scanning surface confocal microscopy, SSCM) and allowed to observe the entry of fluorescently labelled virus like particles (VLPs) into a living cell [71]. By increasing the resolution, even the observation of single VLPs was possible [72]. Furthermore, SSCM allowed to investigate the molecular basis of membrane pits [73], eventually leading to the suggestion of an alternative mechanism for clathrin-coated pit closure [74].

In combination with Förster resonance energy transfer microscopy, SICM revealed that the *β*_2_-adrenergic receptor is redistributed from the transverse tubules in healthy cells to the cell crest in cardiomyocytes from a rat model of heart failure. As a consequence, the corresponding intracellular cyclic adenosine monophosphate signal becomes less confined, suggesting that the redistribution of the *β*_2_-adrenergic receptor might play a role in heart failure [75]. Furthermore, this combination of methods revealed that both the surface structure and the *β*_2_-adrenergic receptor distribution is restored in cells from failing hearts which were treated by genetic methods [76].

The second major types of techniques that have been combined with SICM are electrophysiological recordings. The first experiments with these combined methods applied two glass pipettes. The first one was used for whole cell voltage clamp recording of the cell of interest, while the second one was used to image the topography of the respective cell. By properly adjusting the composition of all electrolyte solutions—the intracellular one (which was accessible by the VC pipette), the bulk solution and the solution within the scanning probe—the scanning probe served as an exclusive potassium ion source. Hence, potassium currents through ATP dependent potassium channels were only recorded by the VC pipette when the scanning probe was in proximity to an active ion channel. This allowed the functional mapping of the corresponding ion channels [77].

In an alternative approach, the SICM probe itself was used for electrophysiological recordings. Firstly, the topography was determined by scanning the probe over the sample. Secondly, the scanning probe was positioned at a membrane structure of interest, was lowered until the probe tip touched the sample and by suction a giga-seal was formed. Then respective voltage protocols were applied to study the ionic current through channels under the probe opening in a cell attached configuration [78]. This technique, which was named *smart patch clamp*, further allowed to spatially determine the neuron-like voltage depended sodium currents in ventricular heart cells [79], to determine the distribution of the maxi-anion channel in cardiomyocytes [80] as well as the clustering of protein kinase A dependent chloride channels in ventricular myoctes [81].

## 4. Molecule Deposition and Electrochemical Analysis

SICM measurements comprise two separated volumes, the bulk solution and the solution within the scanning probe, both connected only by a small interface. Since SICM allows both the determination of the sample topography and the precise control of the tip-sample distance, it has been widely used to deposit certain molecules onto a surface [82–87]. By trapping [88] fluorescent dye molecules in the tip of the probe the probe was applied as a fluorescent nano-sensor [89]. Furthermore, the scanning probe has been used to spatially modify the pH or Na^+^ concentration by the delivery of corresponding ions [90] and has been used to pipet femtoliter-sized droplets [91]. A further application of SICM is the investigation of nano-pores within an artificial membrane [92–95]. Two reviews detail these more chemical applications of SICM [96,97].

Scanning electrochemical microscopy (SECM) is a microscopy technique used to spatially resolve analytes by determining faradayic currents from redox reactions. The close relationship of SICM and SECM has been described in a review of the use of SECM in neuroscience. For more information about SECM the reader is referred to this review and the references therein [98].

To combine SICM and SECM measurements, the scanning probe has to be modified to determine faradayic redox currents. Various probe preparation techniques allowed the simultaneous recording of the topography and an analyte at increasing resolutions [99–103]. It can even be combined with the delivery of molecules through the pipette [104] and used to investigate the transport of redox species through pores in an artificial membrane [105]. Recently, it was applied to detect neurotransmitter release from living hippocampal neurons [106].

### 4.1. Using Double-barrel Probes to Determine the Ion Concentration Surrounding a Living Cell

One method to determine the concentration of an ionic species is the use of ion selective micro-electrodes (ISME) [107–112]. These electrodes consist of a glass micro-pipette the tip of which is filled with an organic ion exchanger resin that forms a complex with a specific species of ions. These carrier molecules are solved in a non-conductive hydrophobic solvent. If this solvent is used as a liquid membrane between two electrolyte solutions containing different concentrations of the ionic species the carrier molecule is able to bind, the bound ion will diffuse through the liquid membrane and will be released on the opposite interface, resulting in a current through the liquid membrane and a potential difference across the membrane will be established according to Nernst's Equation (4). If properly calibrated with solutions of known ion concentration, an ISME can be used to determine an unknown ion concentration of the ion of interest.

**Figure 11.**
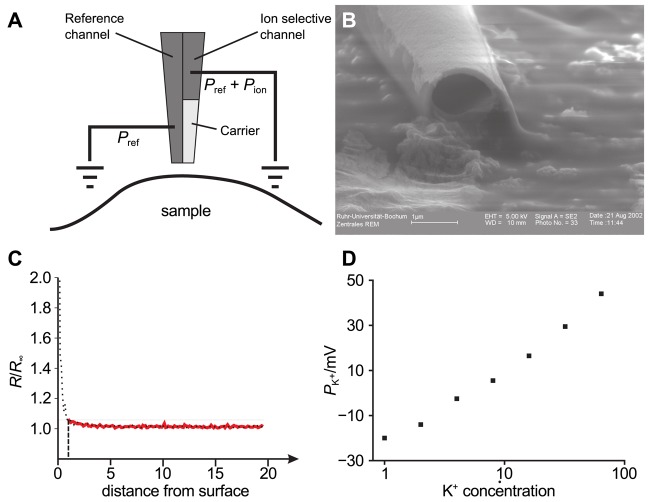
(**A**) Sketch of a double barrel ion selective micro-electrode (ISME). One channel is used as the reference channel and for SICM recording, the tip of the second one is filled with an ion selective liquid membrane, the bulk filled with 100 mM KCl; (**B**) Scanning electron micrographs of a double barrel electrode. Each channel has an opening diameter of approximately 1 *μ*m; (**C**) Approach curve of an ISME towards the cell culture dish. The threshold of 5 % (solid black line) is determined at approximately 1 *μ*m tip-sample distance (dashed line); (**D**) The potential across the liquid membrane follows Nernst's equation.

By using double barrel electrodes, glass pipettes that feature a wall in their center that separates two channels, one channel can be employed for topography determination by SICM and the second one to record the ion concentration of a specific ion species. Figure 11A shows the principle of operation of such a probe used in combined SICM/ISME recordings and Figure 11B shows an electron micrograph of a double barrel electrode with openings of approximately one micrometer.

The probe-sample distance dependence of an ISME is shown in Figure 11C. Here, the location of the surface is approximately reached at a resistance change of 200%. Using this approximation, a resistance increase of 5% approximately indicates a tip-sample distance of one micrometer.

If both channels are filled with the same solution, the potential across the liquid membrane is the sum of the potential of the reference channel and the ion selective potential *E*_ion_. The latter one is described by Nernst's equation:

(4) $$E_{\text{ion}}=-\frac{RT}{zF}\ln\frac{a_{0}}{a_{1}},$$

where *R* is the gas constant, *T* the temperature, *F* is the Faraday constant, *z* is the charge of the ion of interest and *a*_0_ and *a*_1_ are its activities at the opposing sides of the liquid membrane. Figure 11D shows that the potential across the liquid membrane of the ion selective channel of a combined SICM/ISME probe follows Nernst's equation (the investigated ion was K^+^, concentrations were 1, 2, 4, …, 64 mM).

**Figure 12.**
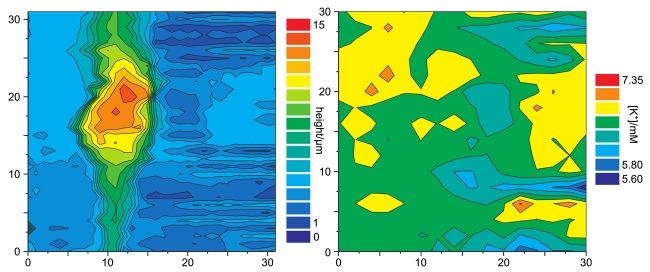
Color coded representations of the topography (left) and the surrounding K^+^ activity (right) of a single cell from rat brain recorded by an ISME. Note, that the reference channel contained 1 mM glutamate to locally stimulate K^+^ efflux.

Figure 12 shows a simultaneous recordings of the topography of a living cell from rat brain cultured on a glia cell layer and the spatially resolved concentration of K^+^. For all experiments shown, a solution of 3mg potassiumtetrakis(4-chlorophenyl)borate in 100*μ*L of 2-nitrophenyl-octyl-ether was used as liquid membrane in the ion selective channel of the SICM/ISME probe. Although the resolution of the potassium concentration mapping is low (since only at every fourth pixel the K^+^ concentration was determined due to the slow response of the ISME), regions of higher K^+^ concentration can be determined near the upper process of the cell as well as at the right side of the image. The latter most likely occurred from an adjacent cell not within the scanning area.

## 5. Conclusions

Here we reviewed the various modes of SICM for studying biological samples. As shown for various examples, the strength of the method particularly resided in its electrical distance control avoiding mechanical damage of the delicate membranes of living cells. We detailed the combinations of SICM with other micro- or nanoscale detection methods such as fluorescence microscopy, patch clamp and ion selective micro-electrodes.

We conclude that although further refinements particularly regarding the temporal resolution are required, that SICM is an emerging technique for life scientists in addition to further potential applications ranging from material sciences to medicine.

## Figures and Tables

**Table 1.** Properties of various SICM imaging modes.

**Mode**	**Speed**	**Sample topography**	**Remarks**
constant *z*	fast	very flat	barely used for imaging topography
DC	fast	flat	fails to detect steep differences in sample height, prone to electrode drifts, likely to contact the sample
AC	fast	moderate	detects intermediate differences in sample height, images at a larger distance
backstep/hopping/STA	slow	arbitrary	best for imaging entire cells and complex sample topography, very low temporal resolution, various enhancements available
FSICM	fast	moderate to complex	hybrid mode combining constant *z*, DC and backstep/hopping/STA mode
